# Yet another new species from one of the best-studied neotropical areas: *Plantago humboldtiana* (Plantaginaceae), an extremely narrow endemic new species from a waterfall in southern Brazil

**DOI:** 10.7717/peerj.2050

**Published:** 2016-05-17

**Authors:** Gustavo Hassemer, Nina Rønsted

**Affiliations:** Statens Naturhistoriske Museum, Københavns Universitet, Copenhagen, Denmark

**Keywords:** Lamiales, Identification key, Neotropics, Plantagineae, Rheophyte, Santa Catarina, South America, Threatened species

## Abstract

This article presents and describes *Plantago humboldtiana*, an extremely narrow endemic rheophytic new species from a waterfall in Corupá, Santa Catarina state, southern Brazil. The new species is unique in presenting a combination of type-G antrorse trichomes on scapes, pendulous inflorescences and 1-seeded pyxidia. Only one population is known to exist, despite intensive search efforts in nearby, similar environments. Its conservation status is assessed as critically endangered (CR) as the only known population is restricted to a dramatically small area, and is subject to extreme fluctuation due to occasional floods, and also to intense visitation by tourists, which can disturb its fragile habitat. We also present an updated identification key to the species of *Plantago* that occur in Santa Catarina. The recent description of three narrow endemic, threatened new species of *Plantago* in Santa Catarina, which is the Brazilian state with its flora best studied, highlights the need for more taxonomic research, especially in the neotropics.

## Introduction

The neotropics harbour around 90,000–110,000 species of seed plants, about 37% of the world’s species, and encompass widely known hotspots for conservation priorities (Antonelli & Sanmartín, 2011). However, a great many plant species in this area are threatened by habitat destruction, overexploitation, and biological invasions. The loss of this biodiversity can have disastrous consequences not only for the environment, but also for humanity (Cardinale et al., 2012; Hooper et al., 2012; Mouillot et al., 2013). Most dramatic is, however, the fact that numerous still undescribed, narrow endemic species may become extinct before they are discovered and described.

*Plantago* L. (Plantaginaceae) is a cosmopolitan genus with about 250 species concentrated in temperate regions and in high-elevation tropical regions (Pilger, 1937; Rahn, 1974; Rahn, 1996; Hassemer et al., 2015; Hassemer, De Giovanni & Trevisan, 2016). Most *Plantago* species have comparatively narrow geographic distributions, many of these being extremely narrow endemics (Dunbar-Co, Wieczorek & Morden, 2008; Meudt, 2012) and/or being threatened with extinction (Rahn, 1974; Hassemer & Baumann, 2014; Hassemer, Baumann & Trevisan, 2014; Hassemer, Trevisan & Rønsted, 2015; Hassemer et al., 2015; Hassemer, De Giovanni & Trevisan, 2016). Some *Plantago* species have a long history of traditional medicinal uses (Samuelsen, 2000), and some new therapeutic and commercially important properties are being discovered (Marlett, Kajs & Fischer, 2000; Fischer et al., 2004; Singh, 2007; Weryszko-Chmielewska et al., 2012).

*Plantago* is comparatively a very well-studied genus, having been the subject of specialised taxonomic treatments by botanists such as Joseph Decaisne (1807–1882), Robert Pilger (1876–1953) and Knud Rahn (1928–2013). Nevertheless, this genus is notable for its complex morphology and taxonomy (Tay et al., 2010b; Meudt, 2011; Hassemer, Trevisan & Rønsted, 2015; Hassemer et al., 2015), which still presents uncertainties regarding species number and circumscription, and the phylogenetic relationships among its sections and species (Rahn, 1996; Ishikawa, Yokoyama & Tsukaya, 2009; Tay et al., 2010a).

**Figure 1:**
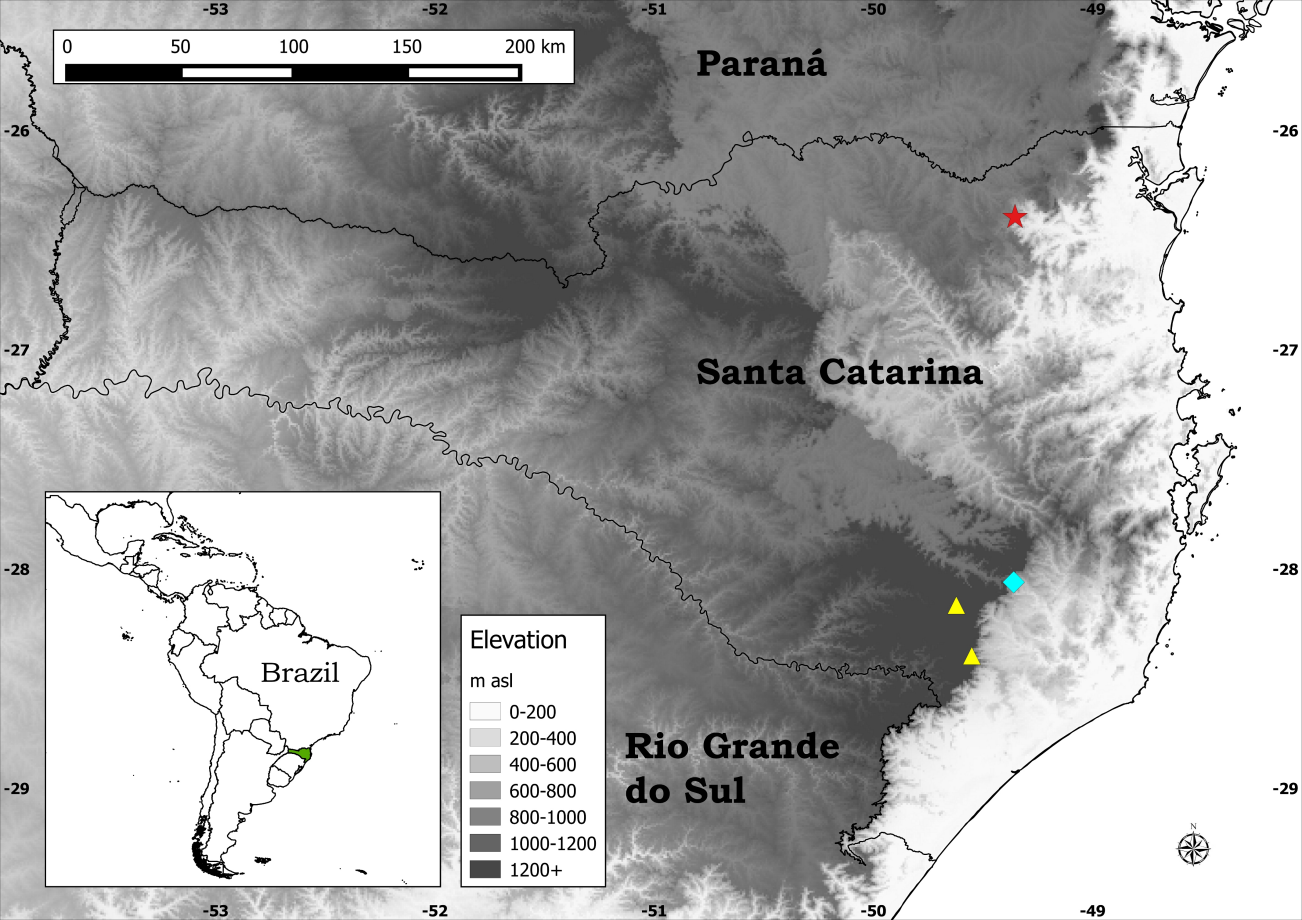
Distribution map of the three species of *Plantago* endemic to Santa Catarina. Legend: Blue diamond, *P. corvensis*; red star, *P. humboldtiana*; yellow triangles, *P. rahniana*.

Despite all previous studies, since 2014 three new species of *Plantago* endemic to Santa Catarina state (SC), southern Brazil, have been described: *P. corvensis* Hassemer (Hassemer & Baumann, 2014), *P. rahniana* Hassemer & R. Trevis. (Hassemer, Baumann & Trevisan, 2014), and the new species being described here (Fig. 1). Additionally, since 2013 six other new plant species endemic to SC have been described: *Sarcoglottis catharinensis* Mancinelli & E.C. Smidt (Mancinelli & Smidt, 2013), *Bothriochloa catharinensis* Dalmolim & A. Zanin (Dalmolim & Zannin, 2014), *Zizaniopsis longhi-wagnerae* Dalmolim et al. (Dalmolim, Zannin & Trevisan, 2015), *Eleocharis guaglianoniana* J.P.R. Ferreira et al. (Ferreira, Venturi & Trevisan, 2015), *Campylocentrum insulare* C.E. Siqueira & E.M. Pessoa (De Siqueira et al., 2015) and *Commelina catharinensis* Hassemer et al. (Hassemer et al., 2016). All of these new species are narrow endemics, having been assessed as endangered (EN) or critically endangered (CR), according to IUCN (2012) and IUCN (2014) criteria.

These discoveries highlight the fact that despite SC being the Brazilian state with the best-studied flora (Reis, Freitas & Cury, 2011; Sousa-Baena, Garcia & Peterson, 2014), there are still many species from this territory to be discovered and described. Furthermore, even for well-studied genera like *Plantago*, in well-studied areas like SC, there are considerable knowledge gaps regarding neotropical biodiversity (Hassemer, Ferreira & Trevisan, 2015; Funez, Hassemer & Trevisan, 2016; Goldenberg et al., 2016), which thwart the implementation of effective policies and measures for the conservation of biodiversity in the neotropics. Additional taxonomic research is a key approach to address the current biodiversity crisis (Agnarsson & Kuntner, 2007; Ebach, Valdecasas & Wheeler, 2011; Wägele et al., 2011; Sluys, 2013), especially in tropical and subtropical areas. Such work should include field work, thorough revision of herbarium material, and state-of-the-art molecular phylogenetic analyses.

This study fills such a knowledge gap by presenting and describing a new, extremely narrow endemic species of *Plantago* from a waterfall in Corupá municipality, Santa Catarina state (SC), southern Brazil, and includes a detailed description, illustrations, and an updated identification key to the species of *Plantago* that occur in SC.

## Materials & Methods

We revised the *Plantago* collections at C, CGMS, DDMS, EFC, FI, FLOR, FT, FURB, GB, HAS, HBR, ICN, MBM, MVFA, MVJB, MVM, PI, SGO, TANG, UFMT and UPCB herbaria (acronyms according to Thiers, 2016). In an attempt to find any other populations of the new species, we searched on two occasions (January 2015 and February 2016) all the 14 waterfalls of the Novo River, which are all located inside the “Reserva Particular do Patrimônio Natural Emílio Fiorentino Battistella” particular environmental protection area in Corupá. The permission to enter and collect plants in this particular area was granted by its director, Reinaldo Langa.

The subgeneric classification of *Plantago* follows Rahn (1978) and Rahn (1996), with the updates of Rønsted et al. (2002), except for *Littorella* P.J. Bergius, which we accept as a genus distinct from *Plantago* (Hoggard et al., 2003). The classification of trichome types follow Rahn (1992). The assessment of the conservation status of the new species followed the IUCN (2012) and IUCN (2014) criteria. Our revised identification key to *Plantago* in SC updates the identification key in Rahn (1966). Unless otherwise stated, all photographs were taken by G Hassemer.

The electronic version of this article in Portable Document Format (PDF) will represent a published work according to the International Code of Nomenclature for algae, fungi, and plants (ICN), and hence the new names contained in the electronic version are effectively published under that Code from the electronic edition alone. In addition, new names contained in this work which have been issued with identifiers by IPNI will eventually be made available to the Global Names Index. The IPNI LSIDs can be resolved and the associated information viewed through any standard web browser by appending the LSID contained in this publication to the prefix “http://ipni.org”. The online version of this work is archived and available from the following digital repositories: PeerJ, PubMed Central, and CLOCKSS.

## Results & Discussion

**Figure 2:**
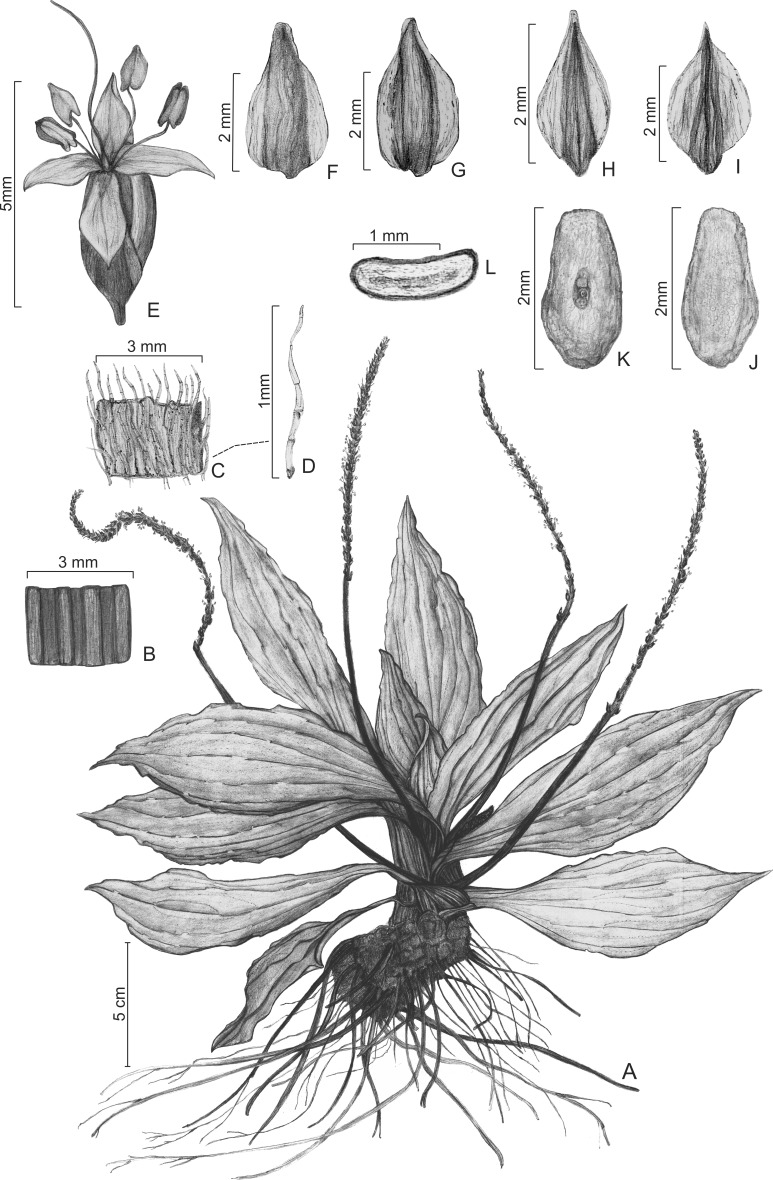
Illustrations of *Plantago humboldtiana*. (A) Habit. (B) Detail of lower part of scape. (C) Detail of upper part of scape. (D) Detail of trichome on scape. (E) Flower. (F) Bract, dorsal face. (G) Bract, ventral face. (H) Anterior sepal, dorsal face. (I) Posterior sepal, dorsal face. (J) Seed, dorsal side. (K) Seed, ventral side. (L) Seed, transversal section. From *G. Hassemer & L.A. Funez 766* (C, M, MBM, WELT); illustrations commissioned from Diogo Chicatto.

**Figure 3:**
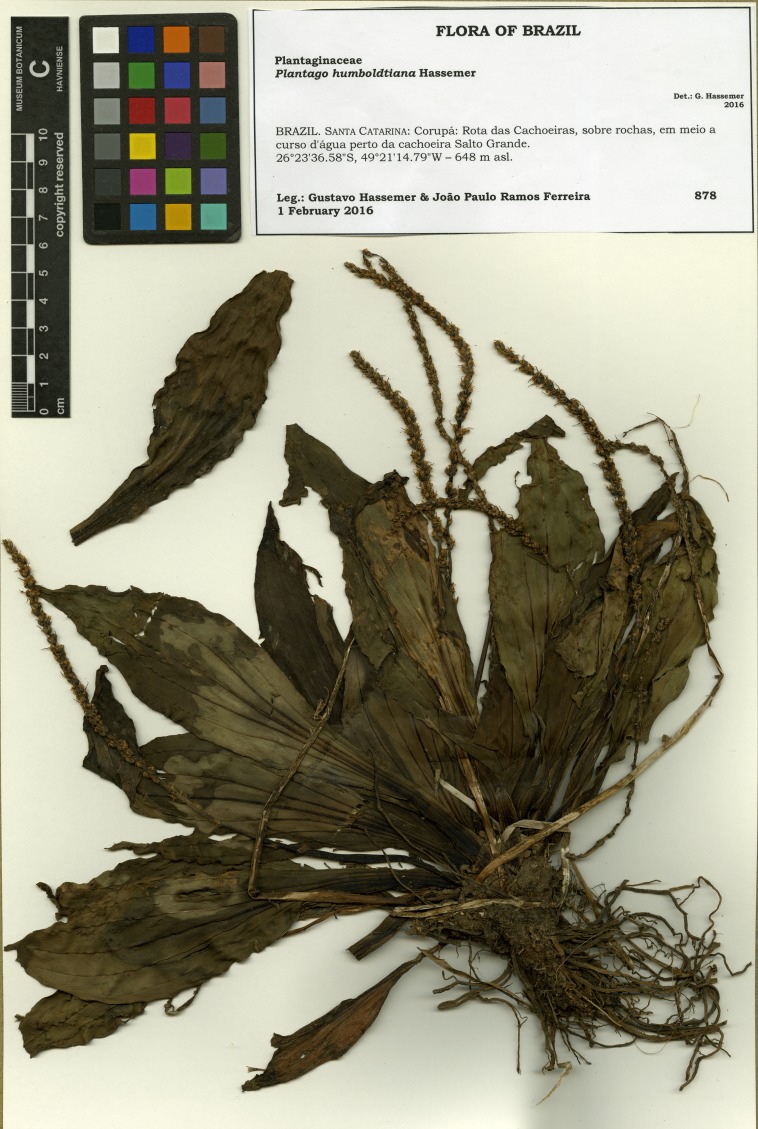
Scanned image of the holotype of *Plantago humboldtiana* (*G. Hassemer & J.P.R. Ferreira 878* (FURB)).

**Figure 4:**
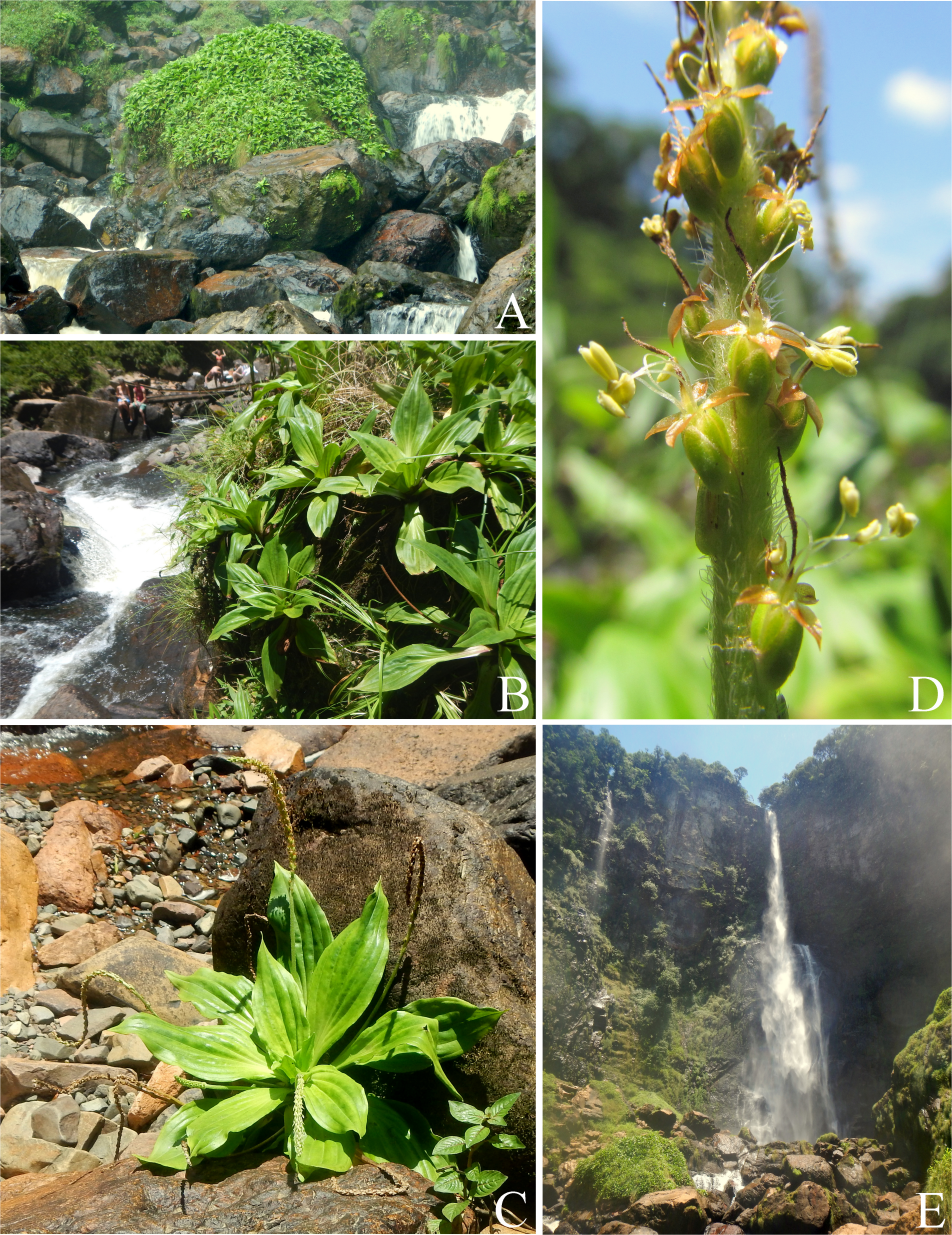
Photographs of *Plantago humboldtiana*. (A) The only known population of *Plantago humboldtiana*. (B) Detail of the environment. (C) Isolated individual clearly displaying the pendulous inflorescences, one of the key characters of this species. (D) Detail of inflorescence (photograph by Luís Adriano Funez). (E) Overview of Salto Grande waterfall, in Corupá municipality, Santa Catarina state.

***Plantago humboldtiana*** Hassemer, sp. nov. (Figs. 2–4).

**Type:** BRAZIL. Santa Catarina: Corupá: Rota das Cachoeiras, sobre rochas, em meio a curso d’água perto da cachoeira Salto Grande, 26°23′36.6″S, 49°21′14.8″W, 1 February 2016, *G. Hassemer & J.P.R. Ferreira 878* (holotype FURB, isotypes B, C, FT, W).

**Paratypes:** BRAZIL: Santa Catarina: Corupá: trilha das cachoeiras, parque municipal, cascata 14, em área de respingos da cascata, ocorrendo sobre rochas e paredões da cascata, 22 April 2014, *R. Trevisan, A. Zannin, A. Reis, S. Venturi, E. Michelena & L. Damazio 1581* (B, C, FLOR); Rota das Cachoeiras, em pedras ao redor da 14^a^ cachoeira, 648 m, 17 January 2015, *G. Hassemer & L.A. Funez 766* (C, M, MBM, WELT).

**Diagnosis:** Leaves lanceolate, glabrous. Trichomes on scapes antrorse, multicellular, eglandular, tape-shaped, flattened, gradually tapering towards the apex and with conspicuous cellular articulations (type G). Inflorescences pendulous on maturity. Flowers less densely distributed in lower part of spike. Sepals asymmetric. Pyxidia 1-seeded.

**Description:** Single-rosette herbs, to 25 cm tall, perennial, darkening considerably on drying. Taproot normally absent, substituted by thickened (2.1–3.3 mm wide) secondary roots. Caudex a short rhizome, 1.0–5.3 × 0.9–3.6 cm, woody. Leaves lanceolate, 9.7–23.0 × 2.8–6.6 cm, 6- to 9-veined, membranaceous, shiny; petiole 0.9–2.2 cm wide, weakly distinct from the lamina; lamina glabrous on both faces; margin entire, glabrous; apex acuminate. Inflorescence 16.3–35.2 cm long, pendulous on maturity. Scapes 11.6–18.4 cm long, cylindrical, generally with longitudinal grooves; trichomes on scapes 0.4–1.1 mm long, tape-shaped (flattened), eglandular, multicellular with very conspicuous cellular articulations, gradually tapering towards the apex (type G), appressed and antrorse, more densely distributed on the upper part of the scape, becoming glabrescent below. Spikes 4.7–16.8 cm long, almost always shorter than the length of the scape, cylindrical, multi-flowered, with flowers rather densely packed above, much less densely crowded below. Bracts triangular, 2.2–2.9 × 1.2–1.9 mm, glabrous to very sparsely ciliate, with rather hyaline wings; apex acute with rounded tip; dorsal face glabrous, rugose; ventral face shiny, glabrous or with very sparsely-distributed long (to 1.4 mm long) trichomes. Flowers hermaphrodite. Anterior sepals elliptic, 1.9–2.3 × 0.9–1.3 mm, glabrous, with rather hyaline wings; apex acute; dorsal face rugose; ventral face shiny. Posterior sepals ovate, 2.2–2.7 × 1.4–1.9 mm, with rather hyaline wings; apex acuminate; dorsal face rugose, glabrous or with very sparsely-distributed long (to 1.1 mm long) trichomes; ventral face shiny, glabrous. Corolla actinomorphic, glabrous; lobes ovate, 1.3–1.8 × 1.1–1.4 mm, shorter than the sepals, patent, rather hyaline, apex acute. Stamens 4; anthers 1.4–1.6 × 0.6–0.8 mm. Ovary with 3 ovules. Pyxidia 2.9–4.7 × 1.8–2.2 mm, 1-seeded. Seed elliptic-ovoid, 2.1–2.9 × 1.3–1.5 mm, surface reticulate, light brown; ventral face slightly concave; dorsal face convex.

**Etymology:** The name is a tribute to the German explorer, geographer and naturalist Friedrich Wilhelm Heinrich Alexander von Humboldt (1769–1859), who had been originally honoured by the name Hansa Humboldt, the original name of Corupá, the Brazilian municipality to which the new species, according to the best of evidence, is restricted. The village of Hansa Humboldt was founded by German immigrants in 1897, and had its original name forcibly changed to Corupá in 1944 due to the Second World War.

**Phenology:** Flowering mainly October–February, fruiting mainly November–March.

**Distribution:** Endemic to a very restricted area (ca. 0.1 km^2^) around Salto Grande waterfall (Fig. 4E), one of the waterfalls of the Novo River, in Corupá municipality, SC, southern Brazil (Fig. 1). Despite thorough search around all the 14 waterfalls of the Novo River on two occasions (January 2015 and February 2016, both during summer, when these plants are flowering and fruiting), not a single individual of *P. humboldtiana* was found outside the area of Salto Grande waterfall.

**Habitat:** Plants of this species grow on rocks adjacent to Salto Grande waterfall (Fig. 4). These rocks are always very humid, due to constant splashing, and are susceptible to occasional flooding, which at times eradicate most plants of this species. However, the species can survive these events, as it was found to be able to recolonise the rocks after a particularly strong flood in 2014.

**Conservation status:** Critically endangered (CR–B2a, c[iv]). This species is restricted to a very narrow area, on a few rocks close to a waterfall (Salto Grande). This locality is included in the “Reserva Particular do Patrimônio Natural Emílio Fiorentino Battistella” particular environmental protection area, but nevertheless is currently threatened by on-going political and economic pressure for the construction of a dam to generate hydroelectric power (A Reis, pers. comm., 2015).

This area is prone to regular flooding, to which this species is apparently well adapted. However, a more important threat to this species is the intense tourist visitation to this waterfall, as we could verify that some visitors climb the rocks around the waterfall, destroying the plants in the process. We strongly recommend that the population of *P. humboldtiana* be protected with a fence and protections signs. Also, the rarity and uniqueness of this species could be positively exploited to raise the awareness among the general public about the biodiversity conservation, and also for environmental education purposes.

Considering all this, we believe this species warrants a critically endangered assessment, and should be the target of *ex-situ* conservation efforts. We further suggest that this species be cultivated in different botanic gardens around the world, and that seeds of this species be collected *in situ* and cryopreserved, in order to permit the re-introduction of this species to its natural environment in the event of its extinction in nature.

**Observations:** *Plantago humboldtiana* is morphologically most similar to *P. australis* Lam. subsp. *australis*, which occurs in southern South America (including southern Brazil); *P. pretoana* (Rahn) Hassemer, which occurs further north in Brazil in Minas Gerais, Paraná and Rio de Janeiro states (Hassemer, Trevisan & Rønsted, 2015; Hassemer et al., 2015); and *P. venturii* Pilg., which is endemic to Tucumán province, northwestern Argentina (Rahn, 1974). However, among these species *P. humboldtiana* is unique and can be promptly distinguished based on its pendulous inflorescences (Fig. 4C) and 1-seeded pyxidia. Even more unique for *P. humboldtiana* is its ecology: no other *Plantago* species worldwide is known to occur in permanently wet rocks amidst waterfalls or rapids (Fig. 4).

According to the infrageneric classification of Rahn (1978) and Rahn (1996), *P. humboldtiana* belongs to *Plantago* sugenus *Plantago* section *Virginica* Barnéoud (Rahn, 1974; Hassemer et al., 2015), based on its characteristically long, wide, stiff, tape-shaped (flattened) eglandular trichomes (type G), its cylindrical, many-flowered spikes, and its asymmetric sepals (Fig. 4D).

Plants of *P. humboldtiana* are striking in their pendulous inflorescences (Fig. 4C), which are a very rare character within *Plantago*. We could observe in many plants that, on maturity, these inflorescences bend and touch the wet stones, in this way allowing the seeds within the pyxidia to germinate and start developing roots while still attached to the mother plants’ inflorescences. This feature allows this species to thrive in otherwise impossible to colonise environments such as rock amidst rapids. The only other species of *Plantago* that has this feature is *P. corvensis*, which is also endemic to SC. However, *P. humboldtiana* differs from *P. corvensis* mainly by its 1-seeded pyxidia, weakly-petiolate leaves and antrorse trichomes on scapes (Fig. 4D), whereas *P. corvensis* has 1–4-seeded pyxidia, distinctly-petiolate leaves and patent trichomes on scapes (Hassemer & Baumann, 2014). Furthermore, *P. humboldtiana* is almost rheophytic, occurring in round and permanently wet rocks amidst rapids in northern SC, whereas *P. corvensis* occurs in vertical rocky cliffs in southern SC (Fig. 1).

### Key to the species of *Plantago* in Santa Catarina state, Brazil

The habitats of each species in SC are presented inside brackets (this is presented in the key only for the individual species). The species not native to SC are marked with an asterisk. This key is an updated version of Rahn (1966).

**Table:** 

1. Scape at least 3.5 times longer than spike. Pyxidia 2-seeded. Seeds deeply concave on ventral side	2
1’. Scape normally shorter or equal to the length of the spike, rarely to 3 times longer in dwarf plants. Pyxidia 1–31-seeded. Seeds slightly concave to slightly convex on ventral side	3
2. Leaves linear. Bract apex obtuse to acuminate. Anterior sepals connate only at base. Corolla zygomorphic, with posterior lobe narrower, and curved at a higher point relative to the other lobes [high-elevation grasslands]	*Plantago brasiliensis* Sims
2’. Leaves lanceolate. Bract apex long cuspidate. Anterior sepals connate for nearly entire length. Corolla actinomorphic [ruderal]	*Plantago lanceolata L.
3. Pyxidia 6–31-seeded. Corolla becoming inconspicuous after fruit maturation. Leaves ovate, with a very evident petiole [ruderal]	*Plantago major L.
3’. Pyxidia 1–4-seeded. Corolla remaining very conspicuous after fruit maturation. Leaves linear to ovate or obovate, with or without a very evident petiole	4
4. Seeds rugose. Trichomes on scapes variously directed, slender, silky, wire-shaped, not very perceptibly tapering towards the apex, with conspicuous cellular articulations. Taproot thickened. Pyxidia 3(–4)-seeded [coastal restingas, high-elevation grasslands, ruderal]	*Plantago tomentosa* Lam.
4’. Seeds reticulate. Trichomes on scapes antrorse, patent or variously directed, stiff or slender, silky or not, wire- or tape-shaped; if wire-shaped and not gradually tapering towards the apex then never with conspicuous cellular articulations. Taproot thickened, unthickened or absent. Pyxidia 1–4-seeded	5
5. Trichomes on leaves and scapes wire-shaped, with inconspicuous cellular articulations, very thin throughout their entire length and not gradually tapering towards the apex (type K)	6
5’. Trichomes on leaves and scapes tape-shaped (flattened), with very conspicuous cellular articulations, gradually tapering towards the apex (type G)	8
6. Leaves elliptic, oblanceolate, or obovate. Pyxidia 2–3-seeded. Caudex globose [high-elevation grasslands]	*Plantago guilleminiana* Decne.
6’. Leaves linear to narrow elliptic. Pyxidia 1–2-seeded. Caudex elongated	7
7. Taproot usually present and thickened, or if absent then substituted by thickened secondary roots. Caudex growing vertically. Leaves linear, with variously-directed trichomes, which never produce a uniformly shiny appearance; the abaxial face with densely distributed, long, silky trichomes, the adaxial face with sparsely distributed, short trichomes [high-elevation grasslands]	*Plantago commersoniana* Decne. ex Barnéoud
7’. Taproot absent, with unthickened secondary roots growing from the caudex. Caudex growing horizontally. Leaves narrow elliptic, with densely distributed, short, antrorse trichomes on both faces, which produces a uniformly shiny appearance [high-elevation grasslands]	*Plantago rahniana*
8. Trichomes on scapes antrorse, generally appressed, but sometimes only very slightly pointing upwards	9
8’. Trichomes on scapes patent	11
9. Inflorescences becoming pendulous on maturity. Pyxidia 1-seeded [rheophytic/rupicolous]	*Plantago humboldtiana*
9’. Inflorescences remaining erect on maturity. Pyxidia 3(–4)-seeded	10
10. Secondary roots to 3 mm wide. Caudex to 3(–5) cm long. Leaves glabrous to glabrescent. Scapes with trichomes concentrated in the upper half, lower half glabrous to glabrescent [high-elevation grasslands, ruderal]	*Plantago australis* subsp. *australis*
10’. Secondary roots to 1.5 mm wide. Caudex to 2 cm long. Leaves pilose. Scapes with trichomes more or less evenly distributed along the entire length [forest edges and clearings, high-elevation grasslands, ruderal]	*Plantago australis* subsp. *hirtella* (Kunth) Rahn
11. Taproot thickened, or absent, then substituted by thickened secondary roots. Leaves ovate, with a very evident petiole. Inflorescences becoming pendulous on maturity [rupicolous]	*Plantago corvensis*
11’. Taproot unthickened, or absent, then substituted by unthickened secondary roots. Leaves narrow lanceolate to oblanceolate or obovate, attenuated, without a very evident petiole. Inflorescences remaining erect on maturity	12
12. Taproot absent. Leaf apices acuminate. Pyxidia 1–2-seeded [high-elevation grasslands]	*Plantago turficola* Rahn
12’. Taproot unthickened. Leaf apices obtuse or acuminate. Pyxidia 3(–4)-seeded	13
13. Caudex very short, generally inconspicuous. Leaves narrowly lanceolate, apex acuminate [coastal restingas, high-elevation grasslands, ruderal]	*Plantago myosuros* Lam.
13’. Caudex elongated and unthickened, very conspicuous in older plants. Leaves oblanceolate to obovate, apex obtuse [coastal restingas, ruderal]	*Plantago catharinea* Decne.

## Additional Information and Declarations

### Field Study Permissions

The following information was supplied relating to field study approvals (i.e., approving body and any reference numbers): The permission to collect plants in the “Reserva Particular do Patrimônio Natural Emílio Fiorentino Battistella” particular environmental protection area in Corupá was granted by its director, Reinaldo Langa.

### Data Availability

The following information was supplied regarding data availability: The research in this article did not generate any raw data.

### New Species Registration

The following information was supplied regarding the registration of a newly described species: *Plantago humboldtiana* Hassemer LSID 77154668-1.

## References

[ref-1] Agnarsson I, Kuntner M (2007). Taxonomy in a changing world: seeking solutions for a science in crisis. Systematic Biology.

[ref-2] Antonelli A, Sanmartín I (2011). Why are there so many plant species in the neotropics?. Taxon.

[ref-3] Cardinale BJ, Duffy JE, González A, Hooper DU, Perrings C, Venail P, Narwani A, Mace GM, Tilman D, Wardle DA, Kinzig AP, Daily GC, Loreau M, Grace JB, Larigauderie A, Srivastava DS, Naeem S (2012). Biodiversity loss and its impact on humanity. Nature.

[ref-4] Dalmolim EB, Zannin A (2014). A new species of *Bothriochloa* (Poaceae, Andropogoneae) endemic to montane grasslands of Santa Catarina, Brazil. Phytotaxa.

[ref-5] Dalmolim EB, Zannin A, Trevisan R (2015). *Zizaniopsis longhi-wagnerae* (Poaceae, Ehrhartoideae), a new grass from montane grasslands of Santa Catarina, Brazil. Systematic Botany.

[ref-6] De Siqueira CE, Pessoa E, Zannin A, Alves M (2015). The smallest angraecoid species from the neotropics: a new *Campylocentrum* (Orchidaceae) from a Brazilian subtropical forest. Systematic Botany.

[ref-7] Dunbar-Co S, Wieczorek AM, Morden CW (2008). Molecular phylogeny and adaptive radiation of the endemic Hawaiian *Plantago* species (Plantaginaceae). American Journal of Botany.

[ref-8] Ebach MC, Valdecasas AG, Wheeler QD (2011). Impediments to taxonomy and users of taxonomy: accessibility and impact evaluation. Cladistics.

[ref-9] Ferreira JPR, Venturi S, Trevisan R (2015). *Eleocharis guaglianoniana* (Cyperaceae), a new species from southern Brazil. Journal of the Torrey Botanical Society.

[ref-10] Fischer MH, Yu N, Gray GR, Ralph J, Anderson L, Marlett JA (2004). The gel-forming polysaccharide of psyllium husk (*Plantago ovata* Forsk). Carbohydrate Research.

[ref-11] Funez LA, Hassemer G, Trevisan R (2016). Rediscovery, typification, and conservation assessment of *Saranthe ustulata* (Marantaceae). Phytotaxa.

[ref-12] Goldenberg R, Michelangeli FA, Aona LYS, Amorim AM (2016). Angiosperms and the Linnean shortfall: three new species from three lineages of Melastomataceae at one spot at the Atlantic Forest. PeerJ.

[ref-13] Hassemer G, Baumann MC (2014). *Plantago corvensis* (Plantaginaceae): a new narrowly endemic species from rocky cliffs in southern Brazil. Journal of the Torrey Botanical Society.

[ref-14] Hassemer G, Baumann MC, Trevisan R (2014). *Plantago rahniana* (Plantaginaceae): a narrow endemic, new species from southern Brazil. Systematic Botany.

[ref-15] Hassemer G, De Giovanni R, Trevisan R (2016). The use of potential distribution models in the study of the distribution and conservation status of plants: the case of *Plantago* L. (Plantaginaceae) in Brazil. Journal of the Torrey Botanical Society.

[ref-16] Hassemer G, Ferreira JPR, Funez LA, Medeiros JD (2016). *Commelina catharinensis* (Commelinaceae): a narrow endemic and endangered new species from Santa Catarina, southern Brazil. Phytotaxa.

[ref-17] Hassemer G, Ferreira PMA, Trevisan R (2015). A review of vascular plant endemisms in Santa Catarina, southern Brazil, highlights critical knowledge gaps and urgent need of conservation efforts. Journal of the Torrey Botanical Society.

[ref-18] Hassemer G, Trevisan R, Meudt HM, Rønsted N (2015). Taxonomic novelties in *Plantago* section *Virginica* (Plantaginaceae) and an updated identification key. Phytotaxa.

[ref-19] Hassemer G, Trevisan R, Rønsted N (2015). Clarifying the occurrence and conservation status of *Plantago dielsiana* Pilg. and *P. australis* Lam. subsp. *pretoana* Rahn (Plantaginaceae) in Brazil. Check List.

[ref-20] Hoggard RK, Kores PJ, Molvray M, Hoggard GD, Broughton DA (2003). Molecular systematics and biogeography of the amphibious genus *Littorella* (Plantaginaceae). American Journal of Botany.

[ref-21] Hooper DU, Adair EC, Cardinale BJ, Byrnes JEK, Hungate BA, Matulich KL, González A, Duffy JE, Gamfeldt L, O’Connor MI (2012). A global synthesis reveals biodiversity loss as a major driver of ecosystem change. Nature.

[ref-22] Ishikawa N, Yokoyama J, Tsukaya H (2009). Molecular evidence of reticulate evolution in the subgenus *Plantago* (Plantaginaceae). American Journal of Botany.

[ref-23] IUCN (2012). IUCN red list categories and criteria.

[ref-24] IUCN (2014). Guidelines for using the IUCN red list categories and criteria.

[ref-25] Mancinelli WS, Smidt EC (2013). *Sarcoglottis catharinensis* (Orchidaceae): a new species from Brazilian Atlantic Forest. Kew Bulletin.

[ref-26] Marlett JA, Kajs TM, Fischer MH (2000). An unfermented gel component of psyllium seed husk promotes laxation as a lubricant in humans. American Journal of Clinical Nutrition.

[ref-27] Meudt HM (2011). Amplified fragment length polymorphism data reveal a history of auto- and allopolyploidy in New Zealand endemic species of *Plantago* (Plantaginaceae): new perspectives on a taxonomically challenging group. International Journal of Plant Sciences.

[ref-28] Meudt HM (2012). A taxonomic revision of native New Zealand *Plantago* (Plantaginaceae). New Zealand Journal of Botany.

[ref-29] Mouillot D, Bellwood DR, Baraloto C, Chave J, Galzin R, Harmelin-Vivien M, Kulbicki M, Lavergne S, Lavorel S, Mouquet N, Paine CET, Renaud J, Thuiller W (2013). Rare species support vulnerable functions in high-diversity ecosystems. PLoS Biology.

[ref-30] Pilger RKF, Engler HGA, Diels FLE (1937). Plantaginaceae. Das Pflanzenreich.

[ref-31] Rahn K, Reitz R (1966). Plantagináceas. Flora ilustrada catarinense. PLAN.

[ref-32] Rahn K (1974). *Plantago* section *Virginica*: a taxonomic revision of a group of American plantains using experimental, taximetric and classical methods. Dansk Botanisk Arkiv.

[ref-33] Rahn K (1978). Nomenclatural changes within the genus *Plantago* L., infraspecific taxa and subdivisions of the genus. Botanisk Tidsskrift.

[ref-34] Rahn K (1992). Trichomes within the Plantaginaceae. Nordic Journal of Botany.

[ref-35] Rahn K (1996). A phylogenetic study of the Plantaginaceae. Botanical Journal of the Linnean Society.

[ref-36] Reis A, Freitas DM, Cury RK (2011). Apresentação das listas das espécies vegetais catarinenses das divisões Angiospermas, Gimnospermas e Pteridófitas. Sellowia.

[ref-37] Rønsted N, Chase MW, Albach DC, Bello MA (2002). Phylogenetic relationships within *Plantago* (Plantaginaceae): evidence from nuclear ribosomal ITS and plastid *trn*L–F sequence data. Botanical Journal of the Linnean Society.

[ref-38] Samuelsen AB (2000). The traditional uses, chemical constituents and biological activities of *Plantago major* L. A review. Journal of Ethnopharmacology.

[ref-39] Singh B (2007). Psyllium as therapeutic and drug delivery agent. International Journal of Pharmaceutics.

[ref-40] Sluys R (2013). The unappreciated, fundamentally analytical nature of taxonomy and the implications for the inventory of biodiversity. Biodiversity and Conservation.

[ref-41] Sousa-Baena MS, Garcia LC, Peterson AT (2014). Completeness of digital accessible knowledge of the plants of Brazil and priorities for survey and inventory. Diversity and Distributions.

[ref-42] Tay ML, Meudt HM, Garnock-Jones PJ, Ritchie PA (2010a). DNA sequences from three genomes reveal multiple long-distance dispersals and non-monophyly of sections in Australasian *Plantago* (Plantaginaceae). Australian Systematic Botany.

[ref-43] Tay ML, Meudt HM, Garnock-Jones PJ, Ritchie PA (2010b). Testing species limits of New Zealand *Plantago* (Plantaginaceae) using internal transcribed spacer (ITS) DNA sequences. New Zealand Journal of Botany.

[ref-44] Thiers B (2016). Index Herbariorum: a global directory of public herbaria and associated staff.

[ref-45] Wägele H, Klussmann-Kolb A, Kuhlmann M, Haszprunar G, Lindberg D, Koch A, Wägele JW (2011). The taxonomist—an endangered race. A practical proposal for its survival. Frontiers in Zoology.

[ref-46] Weryszko-Chmielewska E, Matysik-Woźniak A, Sulborska A, Rejdak R (2012). Commercially important properties of plants of the genus *Plantago*. Acta Agrobotanica.

